# Dynamic chest radiography for wheezing-associated acute respiratory failure: 4 case reports

**DOI:** 10.1016/j.radcr.2025.10.012

**Published:** 2025-11-13

**Authors:** Hidemitsu Miyatake, Masafumi Toshimitu, Shina Nakano, Junji Shimizu, Yasuyuki Tsujita, Kohei Asada, Naoto Shiomi

**Affiliations:** a Department of Emergency and Critical Care Medicine, Shiga University of Medical Science, Otsu, Shiga, Japan; b Department of Emergency and Critical Care Medicine, Kurume University School of Medicine, Kurume, Fukuoka, Japan; c Department of Emergency and Critical Care Medicine, Saiseikai Shiga Hospital, Rittou, Shiga, Japan; d Department of Cardiovascular Medicine, Shiga University of Medical Science, Otsu, Shiga, Japan

**Keywords:** Acute respiratory failure, Wheezing, Dynamic chest radiography, Bedside imaging, Tracheobronchomalacia, Mucus plugging

## Abstract

Dynamic chest radiography is a bedside, low-dose X-ray technique that captures sequential images to visualize respiratory and cardiovascular motion and to derive ventilation maps. We present a case series of 4 patients with wheezing-associated acute respiratory failure from diverse etiologies (presumed asthma exacerbation, tracheobronchomalacia, cardiogenic pulmonary edema, and endotracheal mucus plugging). In each case, dynamic chest radiography provided real-time functional information that complemented initial evaluations when conventional tests were inconclusive. It suggested diffuse airflow limitation in the presumed asthma case, demonstrated expiratory central airway collapse in tracheobronchomalacia, supported pulmonary congestion without ventilation loss in heart failure, and revealed unilateral ventilation impairment due to mucus plugging in an intubated patient. These findings informed bedside clinical decision-making. Dynamic chest radiography may serve as a practical adjunct that increases diagnostic confidence in emergency and critical care settings where rapid, noninvasive functional assessment is desirable.

## Graphical Abstract

**Figure:**
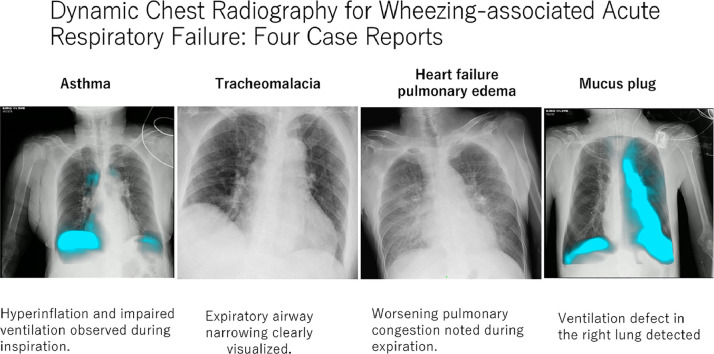

## Introduction

Acute respiratory failure with wheezing is frequently encountered in emergency and critical care, but determining the exact cause can be challenging. Differential diagnoses include cardiogenic pulmonary edema, obstructive pulmonary disease (eg, asthma or chronic obstructive pulmonary disease (COPD) exacerbation), and large airway narrowing. However, conventional tests such as chest radiography or clinical examination alone often fail to confirm the diagnosis promptly, and patients are sometimes diagnosed retrospectively based on treatment response. Although chest radiographs are recommended in acute-onset respiratory failure with wheezing, their diagnostic utility in asthma or COPD exacerbations is limited mainly to excluding alternate pathologies [1]. In such situations, physicians may require additional imaging to avoid diagnostic uncertainty.

Dynamic chest radiography (DCR) has recently been developed as a novel imaging modality that captures sequential chest X-ray images at high frame rates, enabling visualization of thoracic structures in motion over the respiratory and cardiac cycles [2]. Using a portable flat-panel detector system, DCR can acquire chest X-ray images at the bedside, at up to 15 frames per second over a 10-20 second breath-hold or quiet breathing period. The image data are post-processed on a dedicated workstation to generate functional images. One such method is pulsating light mode (PL-MODE), which creates a color-coded lung map that highlights the magnitude of respiratory-induced changes in pixel intensity (ie, lung density) over the breathing cycle. This technique accentuates subtle regional differences in ventilation that are not apparent on static radiographs. For example, lung areas with reduced ventilation exhibit smaller cyclical changes in pixel value and are distinguished visually on the PL-MODE map [3]. Because DCR is performed using a mobile X-ray unit and utilizes low-dose fluoroscopic imaging, it can be performed safely at the bedside even in severely ill or uncooperative patients. This makes it particularly useful in ICU or emergency settings where patients may be unstable or uncooperative. Early studies have shown that DCR can provide useful functional insights. We have previously reported that DCR can aid in the diagnosis of acute pulmonary embolism [4] and in the evaluation of pulmonary congestion due to heart failure [5]. In addition, Robinson et al. have suggested that DCR may be applied to chronic respiratory diseases such as asthma, COPD, and interstitial lung disease [6]. However, reports in acute care settings remain limited, and those specifically addressing acute respiratory failure are particularly scarce. Evidence in emergency and intensive care settings remains confined to small observational studies and isolated case reports; to our knowledge, there are no prospective head-to-head comparisons of DCR against CT, lung ultrasound, or standard chest radiography in patients with acute respiratory failure. These gaps have been highlighted in recent reviews, which also call for larger, standardized studies and bedside protocols for non-cooperative or bedridden patients [6,7]. These illustrative cases were purposefully selected from patients with wheezing-associated acute respiratory failure managed between April and December 2024, in whom DCR was considered particularly useful for diagnosis. In each case, DCR provided visualization of the underlying pathophysiology and aided in the differential diagnosis. All DCR studies were performed at the bedside using a portable X-ray unit equipped with DCR functionality, in the supine position, during quiet breathing for approximately 15 seconds.

## Case presentations

### Case 1

**Fig. 1:**
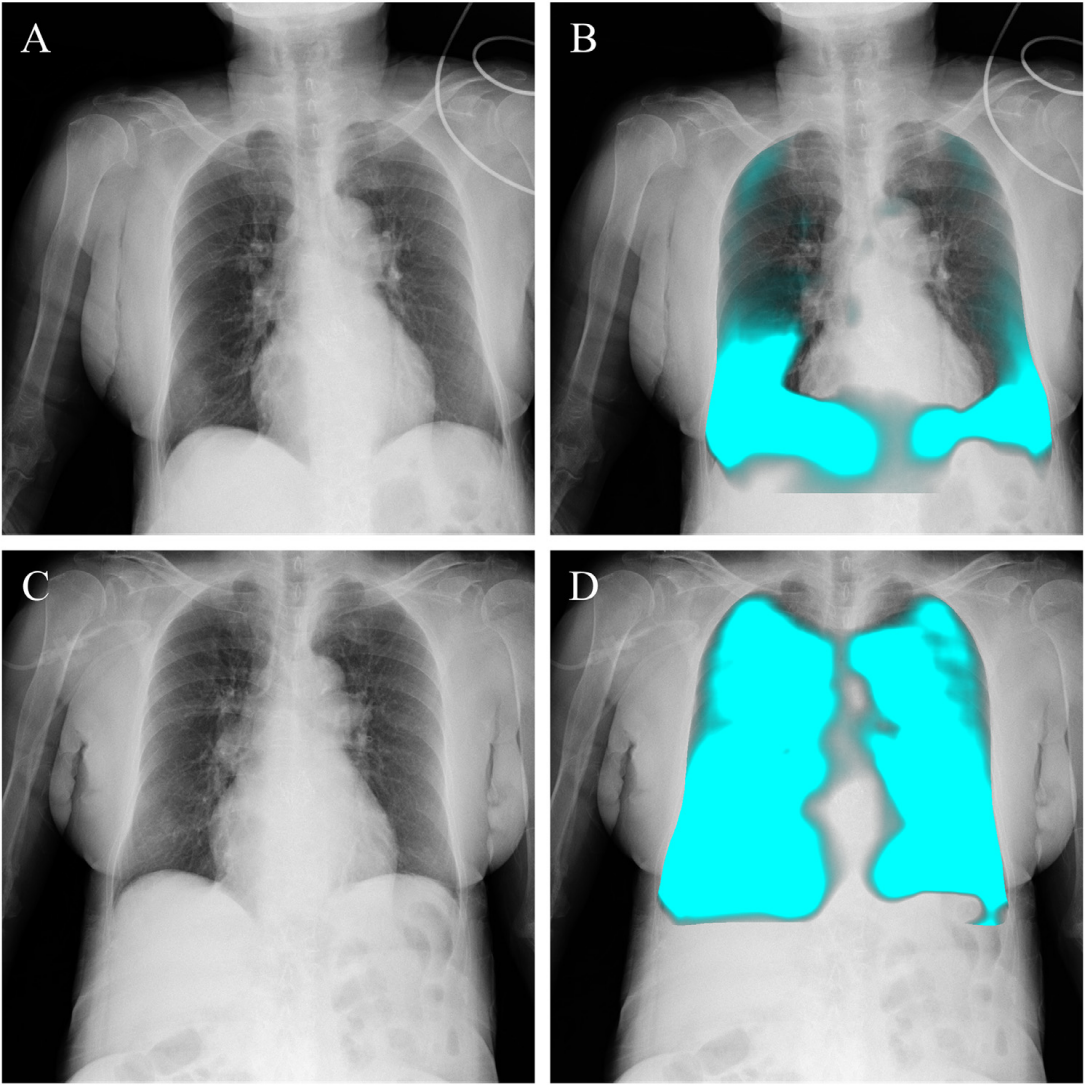
Dynamic chest radiography in a patient with bronchial asthma. The original image (A) and pulsating light mode (PL-MODE) image (B) during the wheezing episode are shown. In the PL-MODE image representing ventilation distribution, a signal reduction is observed throughout both lungs, indicating impaired ventilation. After clinical improvement following bronchodilator and corticosteroid treatment, the original image (C) and PL-MODE image (D) are shown. In the PL-MODE image, signal increase is demonstrated, indicating improved ventilation.

An 81-year-old woman with severe aortic stenosis (ejection fraction 67%) was admitted for elective transcatheter aortic valve implantation (TAVI). She had no history of asthma. The procedure was uneventful, but shortly after extubation she developed acute expiratory wheezing and hypoxemia. A portable chest X-ray showed mild cardiomegaly without overt pulmonary congestion or infiltrates. Bedside DCR revealed increased lung translucency during inspiration, and PL-MODE demonstrated reduced pixel-value fluctuations in both lungs, suggesting impaired ventilation and diffuse bronchospasm (Fig. 1, Supplement videos 1 and 2). After bronchodilator therapy, her wheezing and oxygenation improved within 1 hour. Based on the clinical course and therapeutic response, the episode was diagnosed as an acute asthma exacerbation. Follow-up DCR confirmed recovery of ventilation in both lungs (Fig. 1, Supplement videos 3 and 4).

### Case 2

**Fig. 2:**
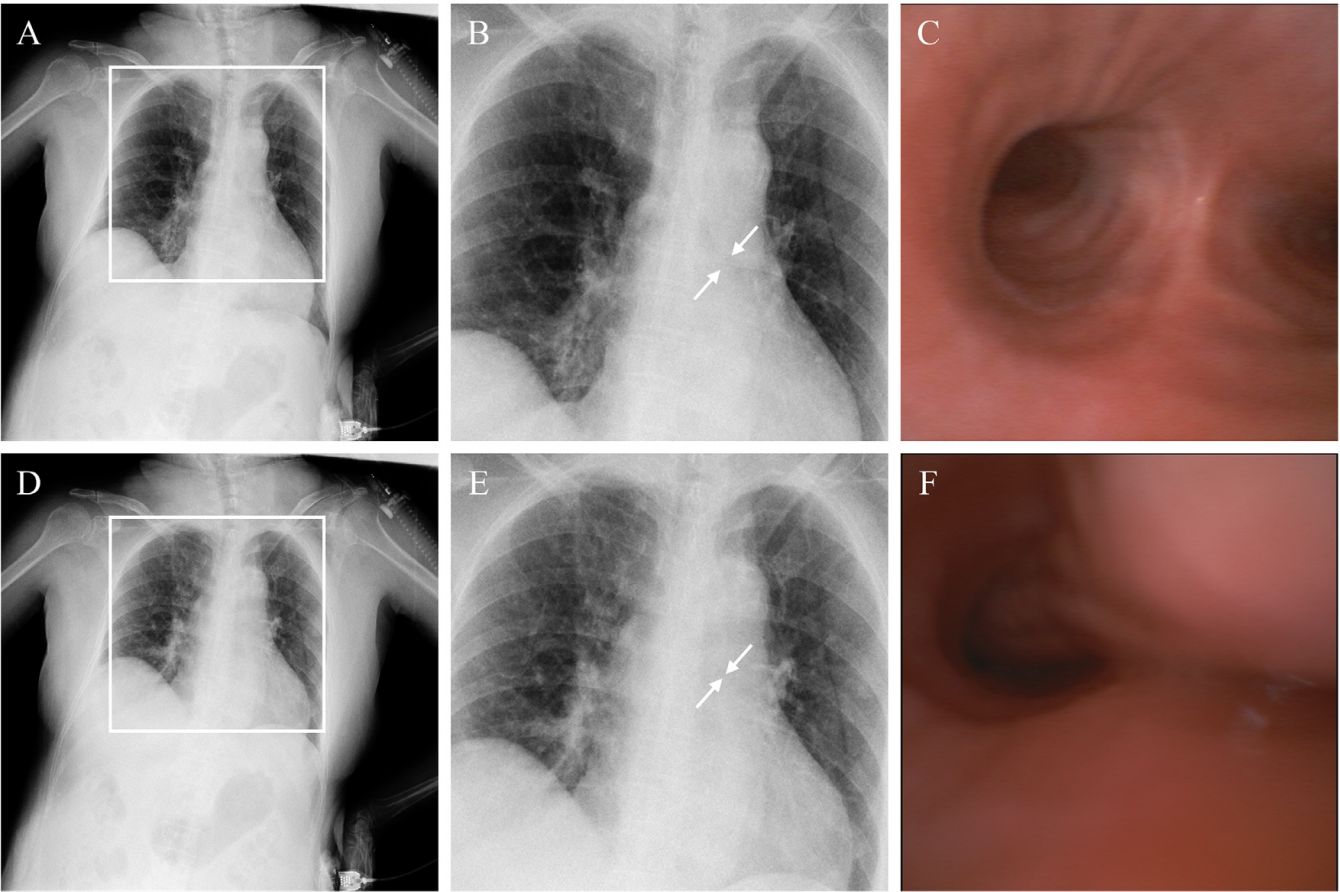
Dynamic chest radiography and bronchoscopic findings in a patient with tracheomalacia. At maximum inspiration, dynamic chest radiography (A) and the magnified chest radiograph (B) clearly show the patency of the left main bronchus (arrow). Bronchoscopy at maximum inspiration (C) also demonstrates patency of both main bronchi. At maximum expiration, dynamic chest radiography (D) and the magnified chest radiograph (E) reveal collapse and obscuration of the left main bronchus (arrow). Bronchoscopy at maximum expiration (F) confirms this collapse. These findings support the diagnosis of tracheobronchomalacia with dynamic airway narrowing during expiration.

A 71-year-old woman with severe dementia, completely dependent in daily activities, had a history of intermittent wheezing that resolved spontaneously. She was brought to the emergency department with new-onset wheezing and hypoxemia after 3 days of fever. On arrival, diffuse expiratory wheezes were audible throughout both lung fields, and oxygen saturation was low. A chest radiograph showed no signs of pulmonary edema or infiltrates. Inhaled β2-agonists and intravenous steroids were administered, but the wheezing persisted. Noninvasive positive pressure ventilation (NPPV) was not feasible due to poor cooperation, and nasal high-flow (NHF) oxygen was initiated, improving oxygenation though wheezing remained. To evaluate central airway patency, chest computed tomography (CT) was performed and revealed narrowing from the distal trachea through the carina into the left main bronchus, raising suspicion of tracheobronchomalacia. Paired inspiratory and expiratory CT is usually recommended, but in this case it could not be performed because the patient’s severe dementia prevented cooperation with respiratory instructions. Therefore, bedside DCR was performed, which demonstrated marked expiratory narrowing of the trachea and left main bronchus in real time, supporting the functional diagnosis of tracheobronchomalacia (Fig. 2, Supplement video 5). Because of persistent respiratory failure, tracheostomy was performed, and bronchoscopy after the procedure corroborated the expiratory airway collapse, leading to a definitive diagnosis of tracheobronchomalacia.

### Case 3

**Fig. 3:**
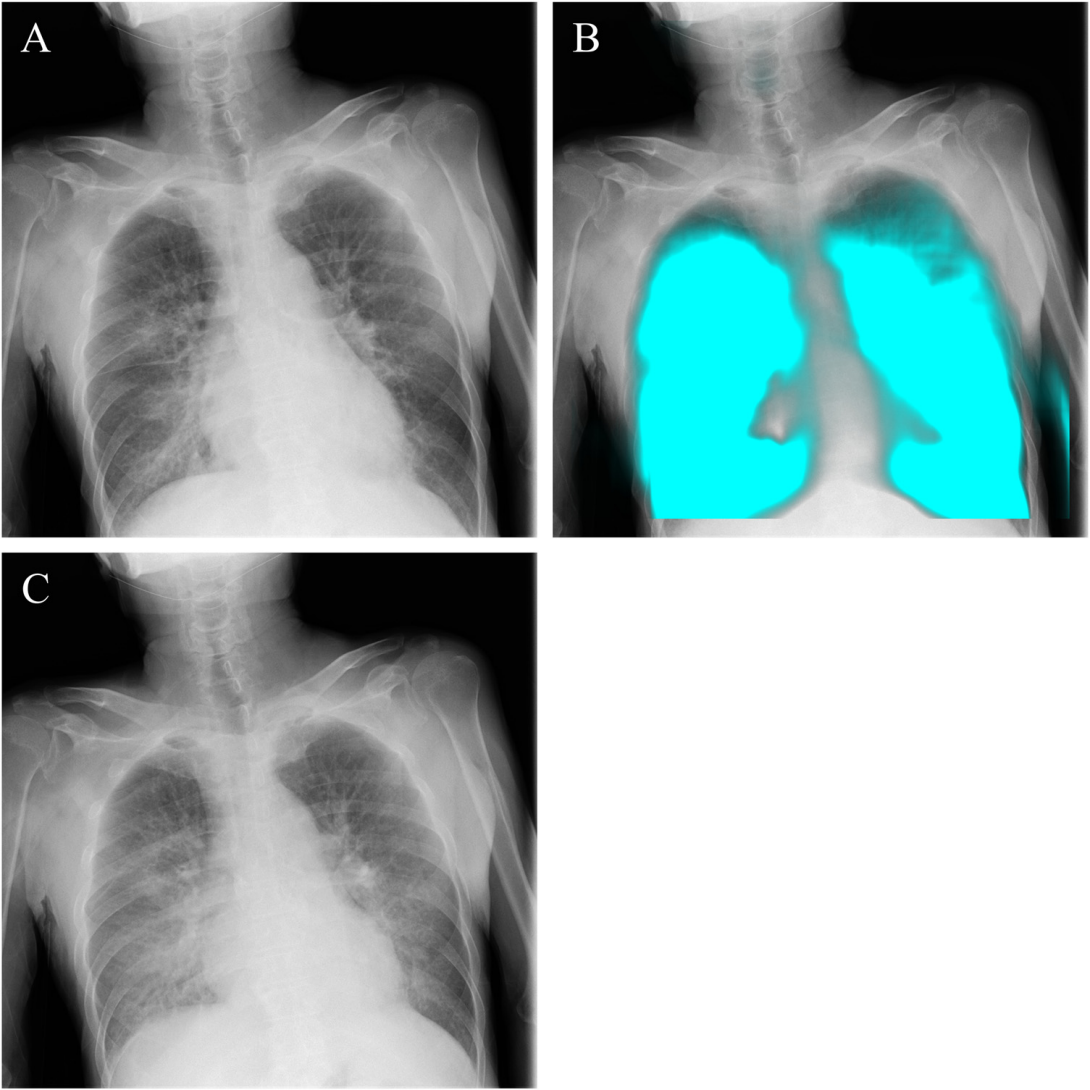
Dynamic chest radiography in a patient with pulmonary congestion. In the original images of dynamic chest radiography, increased lung translucency was observed at maximum inspiration (A), while pulmonary artery dilation and pulmonary congestion were noted at maximum expiration (C). In the PL-MODE image at maximum inspiration (B), a distinct signal increase associated with respiration was observed, suggesting good ventilation.

An 82-year-old man with a history of hypertension, hyperlipidemia, and peripheral arterial disease had experienced dyspnea for about 1 month without treatment. He presented to the emergency department with acute worsening of shortness of breath, accompanied by orthopnea and wheezing. On examination, he had audible expiratory wheezes and mild lower leg edema. A chest X-ray showed mild cardiac enlargement and prominent pulmonary vasculature, which suggested acute heart failure, but a definitive diagnosis could not be made immediately in the emergency setting. Therefore, bedside DCR was performed. DCR demonstrated markedly engorged pulmonary vessels during expiration on dynamic images, while PL-MODE maps showed clear and symmetric respiratory pixel-value fluctuations in both lungs, indicating preserved ventilation (Fig. 3, Supplement videos 6 and 7). These findings suggested that the wheezing was due to pulmonary congestion rather than primary airway obstruction. Subsequent transthoracic echocardiography revealed preserved left ventricular wall motion but elevated pulmonary artery pressure, supporting a diagnosis of cardiogenic pulmonary edema due to acute decompensated heart failure. The patient was treated with noninvasive ventilation, intravenous diuretics, and blood pressure control, leading to improvement in both wheezing and oxygenation.

### Case 4

**Fig. 4:**
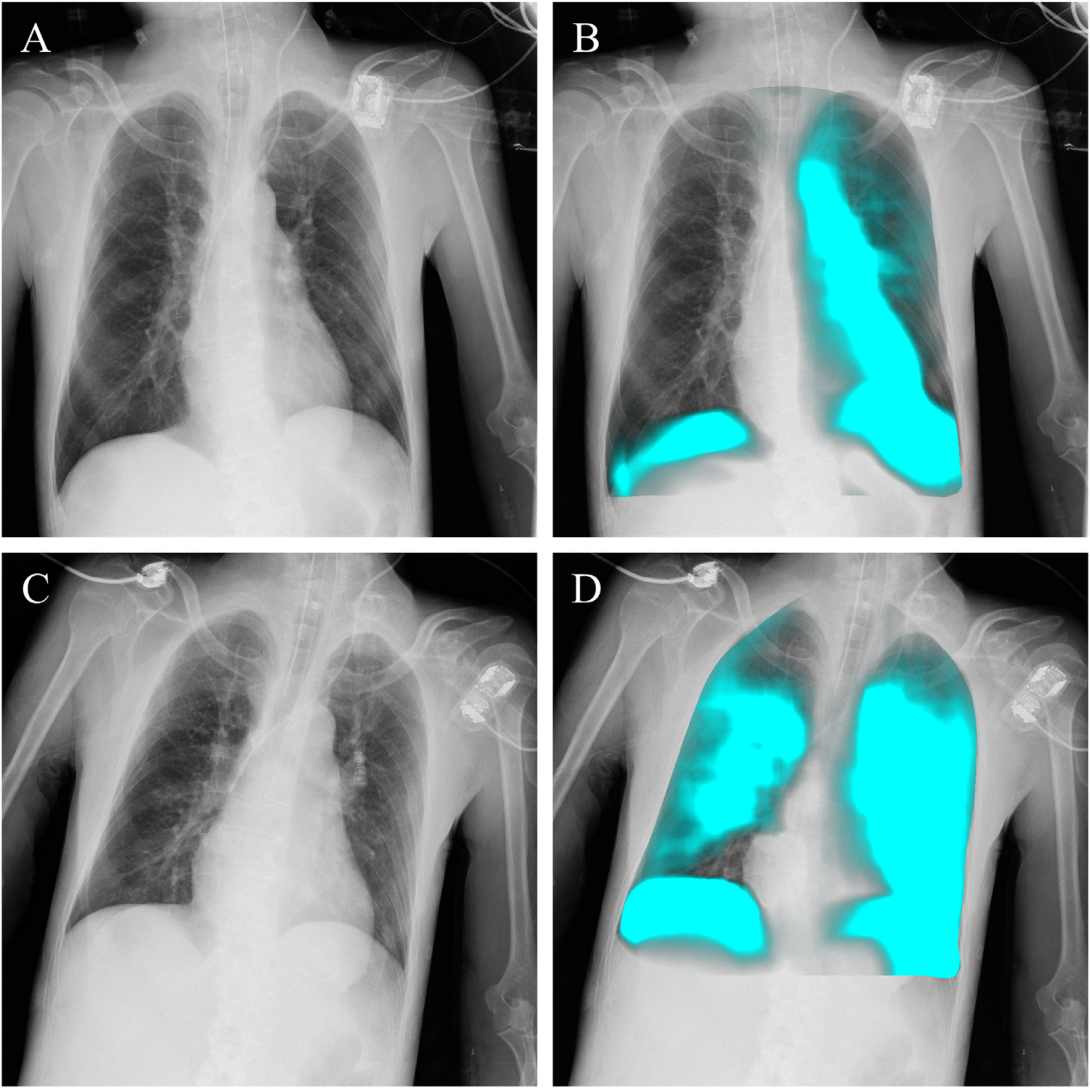
Dynamic chest radiography in a patient with suspected mucus plugging. Dynamic chest radiography during a wheezing episode suspected to be caused by mucus plugging shows the original image (A) and the PL-MODE image (B). In the PL-MODE image, reduced pixel value changes in the right lung were observed, suggesting impaired ventilation. After bronchoscopic suction, clinical improvement was achieved. The follow-up dynamic chest radiography, including the original image (C) and PL-MODE image (D), demonstrated improved ventilation distribution.

A 51-year-old man with no significant past medical history was found collapsed at home and brought to the hospital. On arrival, he was hypothermic, unconscious, and required intubation for airway protection. He was admitted to the intensive care unit (ICU), where rewarming therapy was initiated. Several hours later, expiratory wheezes developed bilaterally, accompanied by decreased lung compliance on the ventilator and worsening oxygenation. A portable chest X-ray showed no findings suggestive of wheezing-related causes such as atelectasis secondary to mucus plugging or pulmonary edema. Bedside DCR was then performed, and PL-MODE imaging demonstrated markedly reduced ventilation in the right lung compared to the left, evident as diminished respiratory pixel-value changes (Fig. 4, Supplement videos 8 and 9). These findings suggested obstruction of the right main bronchus by a mucus plug. Urgent bronchoscopy confirmed and removed a large amount of tenacious mucus obstructing the right bronchial tree. Following suctioning, wheezing resolved and oxygenation improved. A repeat DCR study the next day confirmed restoration of nearly symmetric ventilation, with the right lung showing improved respiratory dynamics (Fig. 4, Supplement videos 10 and 11).

## Discussion

In these 4 cases, DCR provided functional information that supported the diagnosis of diverse causes of acute respiratory failure with wheezing.

Case 1 (Asthma): In acute asthma or chronic obstructive pulmonary disease (COPD) exacerbations, chest radiographs are typically obtained, but their role is mainly to exclude other causes such as pneumonia or pulmonary edema rather than to confirm airflow obstruction [1]. Tests such as peak expiratory flow are often impractical in the acute setting, and diagnoses are usually made retrospectively based on treatment response. In this patient, DCR demonstrated an obstructive ventilatory defect, seen as impaired ventilation and increased lung lucency due to air trapping. These findings did not establish the diagnosis by themselves but provided supportive information that complemented clinical judgment. Compared with plain chest radiography, which was nonspecific in this case, DCR offered dynamic functional evidence that increased diagnostic confidence.

Case 2 (Tracheomalacia): The gold standard for diagnosing tracheobronchomalacia is dynamic airway evaluation, usually by bronchoscopy or paired inspiratory/expiratory CT [8]. In this patient with severe dementia, these approaches were impractical or risky. Chest CT suggested narrowing from the distal trachea to the left main bronchus, but because only a single phase was obtained, it could not demonstrate whether the caliber changed between inspiration and expiration. Thus, CT raised suspicion but could not establish a functional diagnosis. Bedside DCR, however, visualized the expiratory collapse of the trachea and left main bronchus in real time. These findings did not replace bronchoscopy, but provided supportive functional evidence that complemented CT when conventional imaging was inconclusive. Unlike CT, DCR could be performed rapidly at the bedside without patient cooperation, transport, or sedation, highlighting its practicality in uncooperative patients such as those with dementia.

Case 3 (Cardiogenic pulmonary edema): Chest radiographs are commonly used to evaluate pulmonary edema when heart failure is suspected, with high specificity but limited sensitivity [9]. In this patient, the X-ray suggested heart failure but was not definitive in the emergency setting, where rapid decisions must be made at the point of care. DCR provided additional information by visualizing expiratory pulmonary vascular engorgement together with preserved bilateral ventilation on PL-MODE. These findings did not replace chest radiography, but complemented it by reducing diagnostic uncertainty at the bedside. Compared with plain radiographs, which may be subtle and difficult for less experienced clinicians to interpret, DCR offered more intuitive visualization of congestion and ventilation preservation, potentially facilitating earlier recognition of cardiogenic pulmonary edema in emergency care.

Case 4 (Mucus plug): Mucus plugging is a known complication in intubated patients, often causing sudden wheezing and hypoxemia. Chest CT is highly useful for detecting mucus plugs, but chest radiographs rarely demonstrate them unless secondary atelectasis is present [10,11]. In this patient, the portable chest X-ray showed no such findings. Bedside DCR, however, immediately demonstrated a unilateral ventilation defect on PL-MODE, which raised suspicion of a right main bronchial obstruction. Prompt bronchoscopy confirmed and removed a large mucus plug, leading to clinical improvement. These findings did not replace radiography or CT, but provided supportive functional information that helped guide timely intervention. Compared with CT, which requires patient stability and transport, DCR enabled rapid, noninvasive assessment at the bedside, allowing earlier recognition and management of this reversible cause of respiratory failure.

Across these 4 illustrative cases, DCR provided additional bedside functional information that complemented standard chest radiography and helped clarify the underlying pathology in patients with acute respiratory failure and wheezing. The examinations were performed at the bedside, making them feasible regardless of patient condition or cooperation. In particular, PL-MODE functional imaging enhanced detection of impaired ventilation in obstructive processes such as diffuse bronchospasm (case 1) and mucus plugging (case 4). DCR does not replace conventional modalities such as radiography, CT, or bronchoscopy, but contributes supportive dynamic evidence when initial evaluations are inconclusive.

An important practical advantage is that DCR can be performed using a portable X-ray system equipped with dedicated DCR capability, immediately after a standard chest radiograph. If the initial film is inconclusive, an additional 15-second dynamic acquisition can be obtained at the bedside without patient transfer. This workflow enables rapid acquisition of complementary functional information without delaying management, which is particularly valuable in emergency and critical care settings where timely decisions are required. Its advantages include portability, low radiation exposure, and real-time assessment of ventilation and vascular changes, although limitations remain regarding equipment availability and the need for further validation [6,7]. Taken together, these cases suggest that DCR may serve as a useful adjunct in selected emergency and critical care scenarios when conventional radiography alone is insufficient.

## Conclusion

In these 4 cases, DCR provided additional real-time functional information beyond standard chest radiographs and increased diagnostic confidence, particularly when initial evaluations were inconclusive. DCR may serve as a useful tool for evaluating acute respiratory failure with wheezing, particularly when conventional diagnostic tests are inconclusive.

## Patient consent

Written informed consent for publication, including the use of clinical data and relevant images, was obtained from all patients or their legally authorized representatives.

## Appendix Supplementary materials

Video 1. Dynamic chest radiography (original mode) in the acute phase of asthma. Image, video 1

Video 2. Dynamic chest radiography (PL-mode) in the acute phase of asthma. Image, video 2

Video 3. Dynamic chest radiography (original mode) after improvement of asthma. Image, video 3

Video 4. Dynamic chest radiography (PL-mode) after improvement of asthma. Image, video 4

Video 5. Dynamic chest radiography (original mode) in a patient with tracheomalacia. Image, video 5

Video 6. Dynamic chest radiography (original mode) in a patient with congestive heart failure. Image, video 6

Video 7. Dynamic chest radiography (PL-mode) in a patient with congestive heart failure. Image, video 7

Video 8. Dynamic chest radiography (original mode) in a patient with mucus plug obstruction. Image, video 8

Video 9. Dynamic chest radiography (PL-mode) in a patient with mucus plug obstruction. Image, video 9

Video 10. Dynamic chest radiography (original mode) after mucus plug removal. Image, video 10

Video 11. Dynamic chest radiography (PL-mode) after mucus plug removal. Image, video 11
